# Feasibility and Acceptability of a Family-Based Telehealth Intervention for Families Impacted by the Child Welfare System: Formative Mixed Methods Evaluation

**DOI:** 10.2196/57939

**Published:** 2024-10-15

**Authors:** Johanna B Folk, Cynthia Valencia-Ayala, Evan D Holloway, Sarah Anvar, Alison Czopp, Marina Tolou-Shams

**Affiliations:** 1 Department of Psychiatry and Behavioral Sciences School of Medicine University of California, San Francisco San Francisco, CA United States

**Keywords:** family-based intervention, affect management, child welfare system, telehealth, formative evaluation, trauma exposure, substance misuse, adverse childhood experiences, trauma-informed care, adolescent health

## Abstract

**Background:**

Despite elevated rates of trauma exposure, substance misuse, mental health problems, and suicide, systems-impacted teens and their caregivers have limited access to empirically supported behavioral health services. Family-based interventions are the most effective for improving mental health, education, substance use, and delinquency outcomes, yet the familial and placement disruption that occurs during child welfare involvement can interfere with the delivery of family-based interventions.

**Objective:**

To address this gap in access to services, we adapted an in-person, empirically supported, family-based affect management intervention using a trauma-informed lens to be delivered via telehealth to families impacted by the child welfare system (Family Telehealth Project). We describe the intervention adaptation process and an open trial to evaluate its feasibility, acceptability, and impact.

**Methods:**

Adaptations to the in-person, family-based affect management intervention were conducted iteratively with input from youth, caregivers, and systems partners. Through focus groups and collaborative meetings with systems partners, a caregiver-only version of the intervention was also developed. An open trial of the intervention was conducted to assess family perspectives of its acceptability and feasibility and inform further refinements prior to a larger-scale evaluation. Participants included English-speaking families involved in the child welfare system in the past 12 months with teens (aged 12-18 years). Caregivers were eligible to participate either individually (caregivers of origin, kinship caregivers, or foster parents; n=7) or with their teen (caregiver of origin only; n=6 dyads). Participants completed session feedback forms and surveys at pretreatment, posttreatment, and 3-month posttreatment time points. Qualitative exit interviews were conducted with a subset of participants (12/19, 63%) to further understand their experiences with the intervention.

**Results:**

Session attendance was high, and both caregivers and teens reported high acceptability of clinicians and sessions on feedback forms. Families were comfortable with video technology, with very few (<5%) sessions having reported technology problems. Thematic analysis of exit interview transcripts indicated that families used effective communication and affect management skills taught during the intervention. Regarding challenges and barriers, some caregiver-only participants expressed a desire to have their teen also participate in the intervention. All interview participants reported that they would recommend the intervention to others and perceptions of the intervention were overwhelmingly positive. Quantitative surveys revealed differential responses to the intervention regarding affect management and communication.

**Conclusions:**

An open trial of the Family Telehealth Project, a skills-based telehealth intervention for families impacted by the child welfare system, suggests high levels of intervention feasibility and acceptability. Participants noted improvements in areas often hindered by the impacts of trauma and family separation: communication and affect management. Perceptions of the intervention were positive overall for both teens and caregivers. The Family Telehealth Project shows promise in addressing the gaps in behavioral health access for systems-impacted families.

**Trial Registration:**

ClinicalTrials.gov NCT04488523; https://clinicaltrials.gov/study/NCT04488523

## Introduction

### Background

The US child welfare system is comprised of multiple public and private agencies with a mandate to promote the well-being and safety of children. In 2022, over 4 million cases of suspected child abuse or neglect were reported and screened; of those, 3 million met the criteria for further investigation, and 558,899 children were determined to have experienced substantiated child abuse or neglect [1]. Over 200,000 children were removed from their homes, a rate that has gone up precipitously due to the increased number of children who lost a primary or secondary caregiver during the COVID-19 pandemic [2]. Once removed, children are placed in alternative living situations such as a foster family home, group home, or institution; alternatively, fewer than 20,000 were placed in a trial home visit as a precursor to reunification in 2021 [2,3]. At every step of involvement, Black, Latinx, and Indigenous youth are overrepresented in the child welfare system and have been disproportionately impacted by caregiver loss due to COVID-19, with Indigenous and Black youth at the highest risk of foster care placement before the age of 18 years [2,3]. It is important to note that the overrepresentation of ethnoracially minoritized children is not due to the commonly noted “risk factor” of race but is driven by systemic factors including structural racism, cultural bias, and communication barriers (eg, child welfare workers perceiving Black parents to be more hostile in their communication) [4,5].

Both youth and caregivers [6] involved with the child welfare system commonly have extensive trauma histories and are at elevated risk for mental health and substance use concerns [7,8]. Although foster youth receive high rates of mental health services [9], most do not receive or have access to evidence-based care [10,11]. The same is true for caregivers [6], and while having a caregiver with a mental health disorder is associated with less likelihood of family reunification [12], caregivers who receive mental health services have less likelihood of their child entering adoption placement [13].

Family-based interventions are a gold standard for youth impacted by the child welfare system, with demonstrated efficacy in improving mental health, substance use, educational, and delinquency outcomes [14-16]. However, when youth are placed into foster care, geographical distance between family members can interfere with the delivery of family-based interventions [17]. This barrier is of particular concern for teens who are approaching the transition into adulthood and for whom healthy family relationships and support are vital to successful re-entry into their family and community of origin and to improve outcomes into adulthood (eg, secure housing, education and vocational achievement, and mental health treatment engagement, as needed).

Technology holds great promise to mitigate geographical distance barriers between youth and their caregivers, thereby increasing access to family-based interventions. Telehealth (the use of Health Insurance Portability and Accountability Act–compliant videoconferencing tools to provide health services) has been shown to be feasible, acceptable, and efficacious for use in delivering mental health treatment to teens and caregivers, and as part of family-based intervention [18-20], including for underserved populations and when implemented in underresourced settings. Some obstacles in delivering family-based services via telehealth have been described, for example, ensuring family members have access to materials needed in sessions (eg, through mailing material or screen sharing), maintaining fidelity to treatment models, and creating space for individual discussions (eg, related to topics such as suspected family violence) [20]. Conversely, there are many benefits to using technology, particularly as videoconferencing has added new features that can promote creative engagement (eg, using whiteboards, playing web-based video clips, and using visuals in sessions) [20].

Telehealth became more widely used with child welfare–involved families during the COVID-19 pandemic, allowing agencies to offer a broader range of behavioral health interventions [21]. Recent studies of family-based interventions such as Trauma-Focused Cognitive Behavioral Therapy—an empirically supported youth and caregiver intervention to reduce youth traumatic stress—suggest telehealth delivery is feasible, acceptable, and effective in reducing youth trauma symptoms among foster youth and Black youth [22,23]. Given the widespread use of telehealth services, particularly since the onset of the COVID-19 pandemic [24], it is important we build the evidence base regarding how telehealth can promote access to family-based interventions for child welfare–involved families.

### This Study

The goal of this study, the Family Telehealth Project, was to adapt a family-based affect management intervention [25] to be delivered via telehealth and to evaluate its feasibility and acceptability. Intervention content was adapted from the empirically supported intervention, Project RAP-FAMI (Risk reduction for Adolescents and Parents-Family Affect Management Intervention), which was originally designed for English-speaking teens and their caregivers involved in a juvenile drug court program [25]. The goal of the intervention is to reduce youth cannabis use and risk of acquiring sexually transmitted infections (STIs), like HIV, by teaching youth and caregivers the same affect management or emotion regulation skills in order to improve parent-child communication, parental monitoring, and parent-child conflict; family-related constructs associated with youth substance use; and sexual health outcomes.

Based on Emotion Regulation and Social-Personal Frameworks, RAP-FAMI targets four areas of emotion dysregulation: (1) a lack of emotional awareness, (2) inability to tolerate negative emotions, (3) poor behavioral control while experiencing heightened emotion, and (4) limited use of situationally relevant emotion regulation strategies [26,27]. Emotion dysregulation, including negative affect and psychological distress [28], impulsivity [29], and sensation-seeking tendencies [30], is relatively common among systems-impacted youth and associated with more substance use and greater vulnerability to behaviors that increase the risk for HIV and other STIs [28,29]. Emotion dysregulation is a common hallmark of adolescence more generally, and the Social-Personal Framework recognizes adolescence as a period of significant emotional, cognitive, and physical changes. The Social-Personal Framework considers the interplay between individual, social, and environmental influences on teen risk including individual factors, family context, and peer or partner influences. It has shown utility for understanding risk among teens in clinical settings and teens on probation [31,32].

This paper describes the (1) intervention adaptation process for families impacted by the child welfare system, which involved iterative feedback from families and key systems partners; (2) the feasibility of implementing the telehealth intervention during an open trial; and (3) acceptability and impact of the intervention from families’ perspectives.

## Methods

### Family Telehealth Project: Intervention Adaptation Process

The Family Telehealth Project involved the adaptation of the RAP-FAMI [25] for telehealth delivery and to meet the needs of families involved in the child welfare system. The RAP-FAMI intervention focuses on teaching caregivers and youth the same affect management skills (ie, the ability to effectively manage and change the way we feel and cope with various situations) to promote healthier caregiver-youth relationships and a more positive family environment that will thereby improve youth outcomes of relevance (ie, for the population receiving). Core session content therefore includes affect management, parental monitoring, and communication skills (see Table 1 for content by session). The original RAP-FAMI intervention was designed for in-person delivery, with an initial engagement session (focused on barriers to participation in the intervention), 4 core weekly 2-hour sessions, and a fifth 2-hour booster session delivered 3 weeks after the core intervention (total of 11 hours per person). The adapted intervention maintained this structure, although individual and family sessions often happened on different days of the week (or separate weeks, depending on the family’s schedule). In both the original RAP-FAMI and adapted interventions, one clinician meets individually with the youth and another clinician meets individually with the caregiver for 1 hour. During family sessions, both clinicians, the youth, and the caregiver come together for an hour of shared skill-building, practice, and discussion.

Intervention adaptation was conducted iteratively in collaboration with key systems partners serving child welfare–involved families (eg, child welfare supervisors, probation officers, judges, attorneys, and school wellness staff) and through an open trial of the intervention to obtain youth and caregiver perspectives. Before beginning the adaptation process, focus groups were conducted with 19 systems partners, who provided key input regarding existing family-based intervention practices within the California child welfare system; barriers to delivering family-based interventions when youth are placed out of home; and considerations for the current intervention related to session content, technology access, and family engagement [33]. Additional in-depth feedback on the intervention content and implementation was obtained through a 1-time workgroup with 36 attorneys in Northern California and through 4 meetings with representatives from the child welfare, juvenile probation, and court systems. Regarding content, systems partners felt it was important to preserve a focus on substance use and STI risk reduction but also to emphasize areas they believed were most pressing for child welfare–impacted families (eg, trauma, healthy, and unhealthy relationships). Additionally, a need for a caregiver-only version of the intervention was identified for situations when youth are unable to or not interested in participating, or when they are placed out of home in another state and state licensure regulations of study clinicians preclude intervention delivery across state lines. Content topics for the adapted intervention, by session, are displayed in Table 2.

Next, family perspectives on the acceptability of the intervention were obtained through an open trial, which is described below. Youth and caregivers were invited to participate in the full intervention, which included a 1-time engagement session to increase motivation and identify barriers to participation, 4 core intervention sessions, and a booster session. Feedback was iteratively incorporated into the intervention, so participants received slightly different versions of the intervention as the open trial progressed.

**Table 1:** RAP^a^ session content^b^.

Session	Topics			
	Adolescent	Parent	Family	Homework
1	General HIV/STI^c^ information Intro to feelings or affect management	Adolescent sexual development and HIV/STIs Intro to feelings or affect management	Affect management and communication (eg, strengths and weaknesses in communication and monitoring, affect as barrier to effective parent-teen communication, and sexually speciﬁc parent-teen communication)	Practice identifying feelings
2	Managing feelings or the 3Rs^d^ Linking feelings to risk Parent challenge (set-up)	Adolescent development Parental monitoring/ACE IT 3Rs and positive parenting	Parent-teen “RAPping,” part 1: Parent-child communication role-plays and the parent challenge (identifying how teens can be in risk situations and how challenging it can be to stay safe, even for parents)	Parent-teen talk about sensitive topic
3	“RAPping” (assertive communication) with Parents RAPping” with partners or peers about sex risk or drug use HIV/STI risk situations and using affect management (3R) skills	Parental monitoring plans “RAPping” (assertive communication) and using 3Rs with teen “RAPping” (assertive communication) with teen about sex risk or drug use	Parent-teen “RAPping,” part 2: parent-child communication skills practice	Parent: monitoring plan practice; teen: RAP (risk) plan practice
4	Condom use skills Preparing for parent-teen values discussion	Condom use skills Preparing for parent-teen values discussion	Condom use skills activities, family values discussion, family communication plan about risky behaviors that includes affect management (3R) strategies	Family communication plan practice; continued individual parent monitoring and teen RAP risk reduction plan practice
5 (Booster)	Intervention content review Condom skills practice or review Discussion about sex with parent	Intervention content review Condom skills practice or review Discussion about sex with parent	Family communication plan review, condom knowledge and skills	—^e^

^a^RAP: Risk reduction for Adolescents and Parents.

^b^Table published in Tolou-Shams et al [25].

^c^STI: sexually transmitted infection.

^d^3Rs: affect management strategies of remove (and return), release, and reframe.

^e^Not applicable.

**Table 2:** Family telehealth project intervention topics by session and intervention modality (dyadic vs caregiver-only).

Session	Topics			
	Adolescent	Dyadic caregiver	Family	Caregiver only
1	Engagement, reducing barriers to treatment, motivation	Engagement, reducing barriers to treatment, motivation	—^a^	Engagement, reducing barriers to treatment, motivation
2	Rapport building, emotion identification, trauma psychoeducation	Rapport building, emotion identification, trauma psychoeducation	Rapport building, communication game, healthy relationships	Rapport building, emotion identification, trauma psychoeducation
3	Body response to emotions, affect management strategy, substance use, and sex, healthy versus unhealthy relationships	Adolescent development, parental involvement, positive parenting, affect management strategy	Communication styles and role plays	Affect management, effective communication
4	Effective communication, identifying risk situations, risk plan	Effective communication, involvement plan, talking to teens about sex and substance use	Communication styles, observed discussion, and communication coaching session	Adolescent development, parental involvement, involvement plan, talking to teens about sex and substance use
5	Validation	Validation	Validation, family values, communication plan	Validation
6 (Booster)	Intervention content review	Intervention content review	Intervention content review	Intervention content review

^a^Not applicable.

### Ethical Considerations

Ethics approval was obtained from the University of California, San Francisco’s Institutional Review Board (19-28922). Caregiver consent and teen assent were obtained prior to the completion of the baseline assessment and intervention sessions.

Study data do not contain personally identifiable information and are coded with a unique study identification number; a list linking study identification numbers to specific participants is securely maintained and only accessible to trained study staff.

### Participants

Participants included families involved in the child welfare system within the past 12 months with teens (aged 12-18 years) and their caregivers. Caregivers were eligible to participate either individually (caregivers of origin, kinship caregivers, or foster parents; n=7) or with their teen (caregiver of origin only; n=6 dyads). Caregiver of origin refers to the individual or individuals who hold the primary responsibility for the care of the child, typically the birth parent, and does not include those providing temporary care; kinship caregivers refer to individuals caring for a child who is separated from their caregiver of origin and are typically relatives or close family friends; and foster parents are licensed by the state and temporarily providing care for a child separated from their family. Participating caregivers (n=13) were predominately birth parents (n=8, 62%) and on average 43.9 (SD 8.35; range 34-61) years old. Most (n=11, 85%) identified as women. Caregivers identified as American Indian (n=1, 8%), Asian (n=2, 15%), Black (n=4, 31%), Latinx (n=4, 31%), and White (n=3, 23%). Most caregivers (n=10, 77%) reported an annual household income under US $50,000, with an average of 3.23 (SD 1.3) people dependent on this income; 23% (n=3) of caregivers received public assistance and 8% (n=1) of caregivers received disability funds. Of the 6 dyads enrolled, 5 teens participated (1 teen who consented to participate did not complete their baseline assessment or any intervention sessions), and of those, 60% (n=3) identified as girls, 40% (n=2) as boys, and 20% (n=1) as transgender. Teens were on average 14.6 (SD 1.8; range 13-17) years old and identified as Black (n=2, 40%), Latinx (n=2, 40%), multiracial (n=2, 40%), and Native Hawaiian or Pacific Islander (n=1, 20%).

### Procedures

#### Intervention

Caregiver-of-origin participants were given the option to enroll in either the dyadic or individual caregiver version of the intervention, and kinship and foster caregivers were only eligible to complete the individual caregiver intervention. Participants were assigned individual clinicians, who also co-led family sessions in the dyadic condition. Clinicians were licensed clinician employees or unlicensed trainees through the University of California, San Francisco; for 2 dyads, community-based clinicians were trained to serve as the teen clinicians because we expected their already established therapeutic relationships would facilitate session engagement (and trained an entire team within a community-based organization). However, 1 of these teens did not complete a baseline assessment or participate in sessions (described above) and another completed only the engagement session. Sessions were held at the convenience of participants, typically on a weekly basis, during a 3-month intervention period.

#### Quantitative

Youth and caregivers were invited to complete web-based surveys at pretreatment, posttreatment, and 3-month posttreatment time points assessing behavioral health needs and family functioning; compensation was US $50 for each survey completed. Select measures related to the aims of this paper are reported here. Participants were asked to complete feedback forms following each session, which assessed the feasibility and accessibility of session content and clinical facilitation; participants were compensated US $10 for each feedback form completed. In total, each person could earn up to US $250 in the dyadic condition and US $200 in the caregiver-only condition, if they completed all quantitative surveys and feedback forms. Clinicians also completed feedback forms each session to monitor fidelity and identify issues related to the feasibility of sessions.

#### Qualitative

Structured exit interviews were conducted by study staff with caregivers (9/13, 69%) and teens (3/5, 60%). The goal of the exit interview was to understand participants’ experience with and acceptability of the intervention, to see whether they were able to apply what they learned after the intervention, and to identify any barriers or challenges related to the telehealth delivery format or the intervention curriculum. To understand how participants were able to apply the skills taught during the intervention, for example, they were asked to describe a conflict that occurred since completing the intervention and were then asked to describe how they managed the conflict. Participants were asked “What did you discuss regarding this conflict? How did the conversation go?” Their responses to these questions and follow-up questions provided examples of how participants used the skills they learned during the intervention to manage conflict. Although interviewers followed a structured guide with set questions, interviewers were free to ask follow-up questions and probe for details as they deemed necessary. Of the 12 total exit interview participants, 6 (50%) were dyad participants and 6 (50%) were caregiver-only participants. Of the dyads, 1 caregiver and 1 teen from separate families participated while the other member of the dyad did not. Interviews varied widely in length and ranged from 21 to 67 minutes, with a mean length of 38.5 (SD 14.4) minutes. Participants were compensated US $50 for completing the interview.

### Measures

#### Quantitative Measures—Demographics

Participants completed a brief questionnaire assessing demographic characteristics including age, gender, race, ethnicity, and history of involvement with the child welfare system.

#### Clinician Feedback Forms (Feasibility)

Clinicians completed feedback forms each session to identify issues related to the feasibility of covering session activities and whether anything unexpected or adverse occurred during sessions.

#### Youth or Caregiver Session Feedback Forms (Acceptability)

Participants provided feedback on the acceptability of session content including a series of yes or no questions assessing whether they liked the session, whether the topics discussed were important to them, if there were topics that should have been included, and if they would act differently as a result of the session content. Participants also rated the helpfulness of specific intervention activities (1=not at all helpful to 5=very helpful), their clinician (ie, whether they liked their clinician; yes or no; 1=poor to 5=very good on specific clinical skills), and their experience with telehealth technology each session (ie, comfort with technology rated 1=not at all comfortable to 5=very comfortable; whether they experienced any problems with the video technology; yes or no). Forms were completed for all sessions except for the initial engagement session.

#### Difficulties in Emotion Regulation Scale-Short Form

The Difficulties in Emotion Regulation Scale-Short Form [34] is an 18-item measure of the ability to manage emotions. Responses are rated on a scale from 1 (almost never) to 5 (almost always) and averaged to create a total score and 6 three-item subscales: strategies, nonacceptance, impulse, goals, awareness, and clarity. Higher scores reflect greater difficulty with emotion regulation or affect management, with the exception of the awareness subscale items, which were reverse-scored to match the valence of the remaining items.

#### Parent-Teen General Communication Scale

The Parent-Teen General Communication Scale [35] is a 20-item measure of parent-teen communication that yields two subscales: positive (7 items) and negative (13 items) aspects of communication. Statements are rated on a 5-point scale from 1 (never) to 5 (always) and scores are summed to create subscale scores. Higher subscale scores reflect greater positive and negative communication, respectively.

### Data Analysis Plan

#### Qualitative Analysis

Interviews were audio recorded and transcribed verbatim. Qualitative data were analyzed using ATLAS.ti (Lumivero) qualitative data analysis software. Interview transcripts were uploaded into ATLAS.ti, reviewed for accuracy, and then coded by 2 research staffs (CV-A and SA) using thematic analysis, a method for identifying, analyzing, and reporting patterns within data [36]. ATLAS.ti’s analysis function indicated how often codes were identified within the data. Each interview was coded twice, once by each coder. Next, coders reviewed the results and codes collectively and came to an agreement regarding which codes to review for further analysis. The reviewed codes were the codes that appeared most often and those that were related to the research questions.

Effective communication, for example, was coded 69 times throughout the interviews. Interview data were coded as an example of effective communication when participants referenced contact and conversation which led to or contributed to a positive outcome. The tone of voice, choice of words, body language, and other methods of communicating calmly and clearly were recognized as forms of effective communication. As participants discussed the impact of the intervention, impact codes were created. For example, many codes reference intervention impact related directly to communication.

#### Quantitative Analysis

Survey data were collected in REDCap (Research Electronic Data Capture; Vanderbilt University) and managed in SPSS (IBM Corp) and R statistical software (v.4.3.0; R Core Team). Given the pilot study design and small sample size, analyses are all descriptive. Individual trajectories on outcomes of interest were examined using parallel coordinate charts at pretreatment, posttreatment, and 3-month posttreatment time points. Charts were created in R using the *GGally* package’s [37] *ggparcoord()* function. Participant trajectories were grouped by the intervention type (ie, caregiver only vs dyad).

## Results

### Feasibility

Session attendance was high overall. In the individual caregiver version (n=7), 6 (86%) participants completed all possible sessions. In the dyadic version (n=6 dyads), session completion was on average 50% (3/6 possible sessions) for individual caregiver sessions (of the 6 caregivers, 3 completed all individual sessions and 3 completed 0 sessions), 50% (3/6 possible sessions) for individual teen sessions (of the 6 teens, 1 completed all individual sessions, 2 completed 5 sessions, 2 completed 1 session, and 1 completed 0 sessions), and 33% (2/5 possible sessions) for joint sessions (2 dyads attended all joint sessions and the remaining 4 dyads attended 0 joint sessions). Of the 13 participants who provided information about the device used for sessions, participants reported they primarily used phones for sessions (n=9, 69%), with some also using a computer (n=3, 23%) or tablet (n=2, 15%).

Clinicians were largely successful in completing content for core sessions 1 and 4 and the booster; however, they noted some challenges with having sufficient time to complete activities in sessions 2 and 3. In particular, clinicians noted running out of time in a session, in some cases due to starting the session late or needing to spend additional time explaining concepts to certain participants. Given consistent barriers to activity completion, we modified sessions 2 and 3 during the adaptation process to remove activities less central to the hypothesized mechanisms of change; adaptations were iterative, although this change was made toward the end of enrollment (ie, 4 individual caregivers and 1 dyad were enrolled after).

### Acceptability

Caregivers and teens reported high levels of acceptability with sessions and clinicians on session feedback forms. On 59 session feedback forms, caregivers indicated they liked that day’s session 100% (n=59 sessions) of the time, with average ratings of 4.93 (SD 0.31) for how helpful the session was and 4.78 (SD 0.46) for how interesting the session was (scale: 1=not at all to 5=very). These ratings are consistent with teens’ report on 23 feedback forms, who indicated they liked the sessions they attended 100% (n=23 sessions) of the time and rated sessions as helpful (mean 4.04, SD 0.83) and interesting (mean 4.26, SD 0.81). Caregivers (58/59, 98% sessions) and teens (23/23, 100% sessions) reported the topics covered in each session were important to them and 100% liked their clinician. Caregivers and teens both rated clinicians very positively on domains including showing support, listening, answering questions, encouraging discussion, giving information, and keeping the session interesting (scale 1=not at all to 5=very; caregivers average ratings were 4.86-4.90, SDs 0.31-0.43 across domains; teens average ratings were 4.74-5.0, SDs 0.00-0.45 across domains). Both caregivers and teens were very comfortable with video technology (mean 4.85, SD 0.36 and mean 4.26, SD 0.81, respectively; on a scale from 1=not at all to 5=very). Caregivers reported problems with the video technology in only 2 sessions, noting issues with video freezing; teens reported problems with video technology in only 1 session, noting issues with Wi-Fi).

### Qualitative Findings

#### Overview

Exit interviews were conducted with 9 caregivers and 3 teens. All participants responded to each structured interview question and no questions were asked to be skipped or omitted from the data. A number of themes emerged across the interviews during the analysis process.

#### Effective Communication

Caregivers overwhelmingly reported an increase in effective caregiver-youth communication since participating. One caregiver described how conversations with their youth became more effective as a result of participation:

What made it easier is just thinking about it. Because when you’re arguing, and two people are yelling, they’re not hearing each other. They’re just saying what they want to say. And that was the problem. We weren’t hearing each other’s opinions and our point of views. So, we stepped and looked at it from each other’s point of views.

This quote reflects the caregiver’s and teen’s use of affect management skills during a conflict, which helped them bring greater sensitivity and thoughtfulness to their interaction and better understand one another’s opinions. Through practice of these affect management and effective communication skills, caregivers and teens reported they were able to have increasingly productive conversations and empathized with differing points of view.

Participants provided an array of examples of their ability to communicate effectively with family members postintervention. Many spoke about taking space or time to reduce conflict and increase communication. Their responses exemplify both effective communication and the ability to apply the affect management skills they learned to navigate conflict.

Beyond the affect management skill of taking space from the conflict and returning to the topic once in a calmer state, participants discussed their improved communicative abilities in the midst of an argument. When caregivers and youth came back together to manage a conflict, they reported greater empathy toward the other’s position and better understood why they experienced those feelings. This is based on a core intervention skill called the 3 R’s (remove and return, release, and reframe), where participants learn to take space from a heated conflict, use an affect management or cognitive coping strategy, and then return to the conversation.

Although most interview participants were caregivers, 3 teens also participated in the interview process. Regarding how comfortable they felt discussing a conflict with their caregiver, 1 teen participant responded:

Teen Participant: Oh, I was pretty ... at first, I wasn’t that comfortable with it. As I started learning more, it took a different approach. I felt more comfortable.

Interviewer: Yeah. Okay. And how comfortable do you think your mom was talking about this point of conflict?

Teen Participant: I know it was a little uncomfortable for her, too. I feel like it was mutual. But then, as you start using the skills, you’re like, “Okay, we can overcome this.” And we had more confidence talking about it.

The teen articulated the trajectory of feelings they experienced as they attempted to navigate conflict with their caregiver. Their response also speaks to their attunement to their caregiver’s feelings and perspectives. Their ability to represent their caregiver’s disposition is evident in their response, particularly when they stated, “We had more confidence...” as they noted a shared progression.

The ability to communicate effectively is especially crucial for families experiencing placement disruption and child welfare involvement. Participants overwhelmingly reported an increase in their ability to effectively communicate with their caregiver or teen, as well as gratitude for the affect management and parent-child communication skills they gained during the process. Participants were challenged to shift mindsets and behaviors during sessions and were ultimately able to adopt skills that allowed them to engage in challenging conversations to meet resolutions.

#### Affect Management

Families commonly reported that prior to participating in the intervention, they had difficulty regulating their affective responses when conflicts or disagreements occurred. This commonly exacerbated conflict and impeded their ability to reach a resolution. As youth engaged in behaviors that were seen as disagreeable to their caregivers, caregivers reported they would often respond with anger, frustration, and other negative emotions. Thus, affect management was viewed as a valuable tool by caregivers as they reflected on the benefits of the intervention. One teen recalled changes in relationship dynamics:

We didn’t really know how to talk through our problems and get through our problems. And that’s where the program came in and where the Family Telehealth Project was really helpful. It really helped us to learn how to really talk through our problems without letting the tempers flare and cloud our judgment. It taught us how to really step back and put ourselves in each other’s shoes, and to be able to really look at each other’s point of view, without the anger. To stop, and breathe, and look at it, and really try to see where each other’s coming from. And with that, it’s really made a huge difference at where the problem goes after that. It’s really taught us ... it’s really helped diffuse situations in our household and made a huge difference because of that.

Learning to more effectively manage affect led to improvement in both communication and overall relationship building for many families. Affect management was coded by researchers when caregivers reported their ability to manage emotional responses, reflect on how their reactions were impacting their teens or families, and instances of using cognitive reframing (an affect management strategy). The vast majority (8/9, 89%) of caregivers reported an increase in their ability to regulate their affect. As one caregiver reported:

The key thing that I took away from the program is to always just stay calm, cool, collected, and to always be able to see things from the other person’s point of view, from your teen’s point of view. Try to put yourself in their place. Keep yourself calm. Think things through. And if you cannot do that, then you need to remove yourself from the situation and revisit it at another time. That was the key thing I took away.

#### Telehealth Adaptation

Participants were also asked about their experience participating in the intervention over Zoom (a videoconferencing platform). Their responses directly informed the in-person to telehealth intervention adaptation. Given the placement and housing disruptions that disproportionately impact families impacted by child welfare, we were interested in understanding the impact of the telehealth format. Overall, participants reported positive experiences with the telehealth format citing ease, convenience, and ability to build connections.

Overall, participants reported their experience with video-based technology as “smooth,” “very easy,” “good,” and “effective.” All interview participants (n=12) described their experience positively. Although 2 participants reported “one or two glitches” and “dead zones” on their own devices, they described these issues as minor. One participant admitted they were surprised to have no technological or connection issues throughout the intervention.

You know what? The video approach is a lot more convenient. Because when you have kids and other responsibilities, it’s a lot. It’s easier to be home where you can actually look after the kids and other things you need to do in the home and still get the class done.

The participant further reported that aside from a glitch or two, they had no technological issues. In addition to smooth connectivity, participants discussed the convenience of a telehealth option. Participants were able to engage in the intervention in their homes, as they waited in their cars to pick up their children, and when they traveled. They referenced the convenience of not having to sit in traffic or find parking to participate (eg, as they do when receiving services in person at brick-and-mortar agency) and overall seemed to enjoy having the telehealth option.

#### Challenges and Barriers to Treatment

Though participants’ references to communication were overwhelmingly positive as they reflected on the impact of the intervention on their lives, it is important to recognize that the process of implementing new skills was challenging for some. Modifying communication styles and resolving conflict did not come without great effort from families. Participants described how adapting to a new style was not second nature and took practice.

Yeah, because like I said, I’ve tried to consciously do some of the things, but I think it’s slowly somewhat becoming ... not so much second nature right away, but it clicked in a way that starts to become step one every time, instead of remembering after I already flipped out. “Oh, yeah, I’m supposed to do this other thing first.

This was a common sentiment among participants, who noted while they practiced the communication skills they learned, it took time before they started to feel more natural. For many, the Family Telehealth Project was the first time they were introduced to skills related to communication, and the application of new knowledge to their real lives was more of a challenge for some than others. Given the short intervention time frame and the number of skills taught, participants tended to forget skills or revert back to previous behaviors before successfully implementing skills they had learned. Despite these challenges, participants explained that every situation of skills practice improved their ability to apply them the next time.

Participants were also asked about their least favorite part of the intervention or things they would change. Some participants reported no concerns about the program and continued to report their satisfaction with the services they received. Some caregivers who participated in the intervention individually reported disappointment that their teen did not participate with them.

But the least favorite thing was the not being able to do it together. That was the only problem that I had with the program. I really would’ve liked to, you know, have it one-on-one. And even if we couldn’t come in, you know, together. I think that he really would’ve benefitted because it would’ve been one-on-one.

### Quantitative

**Figure 1:**
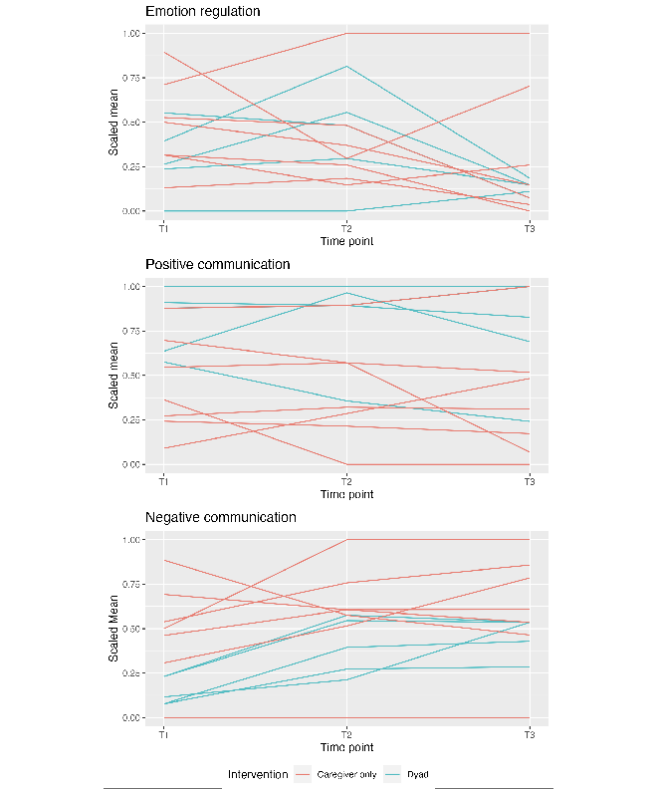
Parallel coordinate charts for main outcomes stratified by intervention type. Scaled means were used because each measure is on a different scale. Minimum scores were scaled to 0 and maximum scores were scaled to 1. T1: preintervention time point; T2: postintervention time point; T3: 3-month postintervention time point.

Parallel coordinate charts are included in Figure 1. For both intervention types, from the preintervention to postintervention time point, most participants (8/12, 66.7%) reported similar amounts of challenges regulating emotions; 3 (25%) reported increased challenges regulating emotions, including 2 dyad participants, while 1 caregiver-only participant reported a sharp decrease in challenges regulating emotions. From the postintervention to 3-month postintervention time point, most participants (7/12, 58.3%) saw similar or fewer challenges regulating their emotions; 1 dyad participant saw a sharp increase, but it was still lower than their preintervention level, while another stayed consistently higher than the rest of the sample.

For both intervention types, half of participants (6/12, 50%) reported similar levels of positive communication across time points. One caregiver-only participant reported a steady increase from the preintervention to 3-month postintervention time point. Two caregiver-only participants and 1 dyad reported decreases in positive communication from the preintervention to 3-month postintervention time point. For caregiver-only participants, there were mixed results for negative communication. All dyad participants (5/5, 100%) reported slight increases in negative communication from the preintervention to postintervention time point, and those remained constant or slightly increased to the 3-month postintervention time point.

## Discussion

### Principal Findings

Initial findings of a family-based affect management intervention delivered via telehealth (the Family Telehealth Project) to teens and caregivers impacted by the child welfare system indicate both teens and caregivers find such intervention to be highly feasible and acceptable. Session attendance was high, and both caregivers and teens reported they felt very comfortable with the video technology. Issues with technology were extremely rare, and satisfaction with video-based services was high. Families reported the telehealth modality reduced previously experienced barriers to attendance such as parking, traffic, drive time, and childcare. The telehealth option also made it possible for teens who lived outside of (and often far away from) their caregivers to participate in a family-based intervention. Qualitative responses suggested participants found the intervention to be helpful and indicated an overall positive experience with the telehealth format.

Caregivers and teens described the learning and practice of affect management and communication skills as highly beneficial. However, qualitative and quantitative responses were somewhat discrepant regarding changes in participants’ ability to communicate effectively and manage their emotions. During qualitative exit interviews, participants expressed they began to consider their family members’ perspectives, pause prior to or during an escalation to reduce conflict, communicate emotions, and identify their own emotions in order to practice affect management. Quantitative survey data did not consistently reflect improvement, however, with some respondents reporting no change or increased difficulties. One possibility is those reporting increased difficulties had actually built more insight into their own affect management and communication patterns. Alternatively, those who perceived the intervention to be more beneficial may have been more willing to participate in the exit interview (12/19, 63% of participants completed an exit interview). The small sample size precluded statistical analysis to quantify changes and differential intervention response; this will be explored in future analysis with a larger sample. The discordant findings suggest the importance of using mixed methods to understand participants’ experiences. Their self-reported improvement in using affect management skills expressed in exit interviews speaks to the perceived benefits of the intervention both from personal and familial frames and suggests the need for further investigation into the interventions’ effectiveness.

### Strengths, Limitations, and Future Directions

This study addresses an important gap in our understanding of how to serve youth and families impacted by the child welfare system through leveraging technology. Despite the overwhelming positive feedback from participants, even from those who shared concerns, it is important to identify any potential barriers to the most effective treatment to improve services and outcomes. Our process of adapting an in-person intervention to telehealth involved multidisciplinary systems professionals [33] and iterative input from youth and caregivers, resulting in intervention families found highly acceptable and impactful. One limitation is our small sample size, which allowed us to reach saturation for qualitative analysis but was insufficiently powered for conducting quantitative analyses; this will be addressed subsequent to the completion of a larger trial of the intervention. Relatedly, the majority of caregivers participated in the individual version of the intervention rather than the dyadic version, although we were insufficiently powered to compare the two versions to understand differential effectiveness. Outcome analyses with a larger sample are needed to understand whether the 2 versions are comparable in terms of effectiveness or whether dyadic skills learning and practice are necessary. Additionally, attendance at dyadic sessions was bimodal, with some families attending all possible dyadic sessions and others attending none; this may indicate a need to consider even briefer approaches for some families such as single-session family consultation models [38].

### Conclusions

Families impacted by the child welfare system participated in a telehealth intervention, which they found to be highly feasible and acceptable. Families were receptive to learning affect management and communication skills over telehealth, indicating the modality helped reduce barriers to care and that they experienced few technology challenges. Additional data regarding the impact of participation on behavioral health and family functioning outcomes are needed; however, qualitative insights are sufficiently promising to move to the next stage of research (ie, larger trial). The use of telehealth for skills-based family interventions with this population has the potential to reduce barriers to service access and support families in remaining connected and strengthening their relationships while separated.

## Abbreviations

RAP-FAMI: Risk reduction for Adolescents and Parents-Family Affect Management Intervention
REDCap: Research Electronic Data Capture
STI: sexually transmitted infection

## References

[1] (2024). Child maltreatment 2022. Children's Bureau.

[2] Hillis SD, Blenkinsop A, Villaveces A, Annor FB, Liburd L, Massetti GM, Demissie Z, Mercy JA, Nelson Iii CA, Cluver L, Flaxman S, Sherr L, Donnelly CA, Ratmann O, Unwin HJT (2021). COVID-19-associated orphanhood and caregiver death in the United States. Pediatrics.

[3] (2023). Child maltreatment 2021. Children's Bureau.

[4] Cénat JM, McIntee SE, Mukunzi JN, Noorishad PG (2021). Overrepresentation of black children in the child welfare system: a systematic review to understand and better act. Child Youth Serv Rev.

[5] Dettlaff AJ, Rycraft JR (2010). Factors contributing to disproportionality in the child welfare system: views from the legal community. Soc Work.

[6] Cheng TC, Lo CC (2020). Mental health services receipt among caregivers in the child welfare system: a longitudinal analysis. Child Youth Serv Rev.

[7] Blakey JM, Hatcher SS (2013). Trauma and substance abuse among child welfare involved African American mothers: a case study. J Public Child Welfare.

[8] Marcenko MO, Lyons SJ, Courtney M (2011). Mothers' experiences, resources and needs: the context for reunification. Child Youth Serv Rev.

[9] Horwitz SM, Hurlburt MS, Heneghan A, Zhang J, Rolls-Reutz J, Fisher E, Landsverk J, Stein RE (2012). Mental health problems in young children investigated by U.S. child welfare agencies. J Am Acad Child Adolesc Psychiatry.

[10] Horwitz SM, Chamberlain P, Landsverk J, Mullican C (2010). Improving the mental health of children in child welfare through the implementation of evidence-based parenting interventions. Adm Policy Ment Health.

[11] Aarons GA, Palinkas LA (2007). Implementation of evidence-based practice in child welfare: service provider perspectives. Adm Policy Ment Health.

[12] Marshall JM, Huang H, Ryan JP (2011). Intergenerational families in child welfare: assessing needs and estimating permanency. Child Youth Serv Rev.

[13] Cheng TC, Li AX (2018). Maltreatment and families’ receipt of services: associations with reunification, kinship care, and adoption. Fam Soc.

[14] Leve LD, Fisher PA, Chamberlain P (2009). Multidimensional treatment foster care as a preventive intervention to promote resiliency among youth in the child welfare system. J Pers.

[15] Henggeler SW (2012). Multisystemic therapy: clinical foundations and research outcomes. Psychosocial Intervention.

[16] Diamond G, Josephson A (2005). Family-based treatment research: a 10-year update. J Am Acad Child Adolesc Psychiatry.

[17] Schneiderman JU, McDaniel D, Xie B, Arnold Clark JS (2010). Child welfare caregivers: an evaluation of access to pediatric health care. Child Youth Serv Rev.

[18] Greenwood H, Krzyzaniak N, Peiris R, Clark J, Scott AM, Cardona M, Griffith R, Glasziou P (2022). Telehealth versus face-to-face psychotherapy for less common mental health conditions: systematic review and meta-analysis of randomized controlled trials. JMIR Ment Health.

[19] McLean SA, Booth AT, Schnabel A, Wright BJ, Painter FL, McIntosh JE (2021). Exploring the efficacy of telehealth for family therapy through systematic, meta-analytic, and qualitative evidence. Clin Child Fam Psychol Rev.

[20] Robbins MS, Midouhas H (2021). Adapting the delivery of functional family therapy around the world during a global pandemic. Glob Implement Res Appl.

[21] Coon JC, Bush H, Rapp JT (2022). Eight months of telehealth for a state-funded project in foster care and related services: progress made and lessons learned. Behav Anal Pract.

[22] Martin AN, McLeigh JD, Lamminen LM (2023). Examining the feasibility of telehealth trauma-focused cognitive behavioural therapy (TF-CBT) with young people in foster care. J Child Adolesc Trauma.

[23] Burrow-Sánchez JJ, Hops H (2019). A randomized trial of culturally accommodated versus standard group treatment for Latina/o adolescents with substance use disorders: posttreatment through 12-month outcomes. Cultur Divers Ethnic Minor Psychol.

[24] Molfenter T, Roget N, Chaple M, Behlman S, Cody O, Hartzler B, Johnson E, Nichols M, Stilen P, Becker S (2021). Use of telehealth in substance use disorder services during and after COVID-19: online survey study. JMIR Ment Health.

[25] Tolou-Shams M, Dauria E, Conrad SM, Kemp K, Johnson S, Brown LK (2017). Outcomes of a family-based HIV prevention intervention for substance using juvenile offenders. J Subst Abuse Treat.

[26] Gratz KL, Roemer L (2004). Multidimensional assessment of emotion regulation and dysregulation: development, factor structure, and initial validation of the difficulties in emotion regulation scale. J Psychopathol Behav Assess.

[27] Steinka-Fry KT, Tanner-Smith EE, Dakof GA, Henderson C (2017). Culturally sensitive substance use treatment for racial/ethnic minority youth: a meta-analytic review. J Subst Abuse Treat.

[28] Lucenko BA, Malow RM, Sanchez-Martinez M, Jennings T, Dévieux JG (2003). Negative affect and HIV risk in alcohol and other drug (AOD) abusing adolescent offenders. J Child Adolesc Subst Abuse.

[29] Dévieux J, Malow R, Stein JA, Jennings TE, Lucenko BA, Averhart C, Kalichman S (2002). Impulsivity and HIV risk among adjudicated alcohol- and other drug-abusing adolescent offenders. AIDS Educ Prev.

[30] Bryan A, Stallings MC (2002). A case control study of adolescent risky sexual behavior and its relationship to personality dimensions, conduct disorder, and substance use. J Youth Adolesc.

[31] Donenberg G, Emerson E, Kendall AD (2018). HIV-risk reduction intervention for juvenile offenders on probation: the PHAT Life group randomized controlled trial. Health Psychol.

[32] Brown LK, Hadley W, Donenberg GR, DiClemente RJ, Lescano C, Lang DM, Crosby R, Barker D, Oster D (2014). Project STYLE: a multisite RCT for HIV prevention among youths in mental health treatment. Psychiatr Serv.

[33] Leo HP, Folk JB, Rodriguez C, Tolou-Shams M (2023). Implementation considerations for family-based telehealth interventions for youth in foster care: focus group study with child welfare system professionals. JMIR Form Res.

[34] Kaufman EA, Xia M, Fosco G, Yaptangco M, Skidmore CR, Crowell SE (2015). The Difficulties in Emotion Regulation Scale Short Form (DERS-SF): validation and replication in adolescent and adult samples. J Psychopathol Behav Assess.

[35] Barnes HL, Olson DH (1985). Parent-adolescent communication and the circumplex model. Child Dev.

[36] Braun V, Clarke V (2006). Using thematic analysis in psychology. Qual Res Psychol.

[37] Schloeke B, Cook D, Larmarange J, Briatte F, Thoen E, Elberg A, Toomet O, Crowley J (2024). GGally: extension to 'ggplot2'.

[38] Hoyt MF, Young J, Rycroft P (2020). Single session thinking 2020. ANZ J Fam Ther.

